# Radiosensitization of hypoxic tumour cells by *S*-nitroso-*N*-acetylpenicillamine implicates a bioreductive mechanism of nitric oxide generation

**DOI:** 10.1038/sj.bjc.6690173

**Published:** 1999-03

**Authors:** M Y Janssens, V N Verovski, D L Van den Berge, C Monsaert, G A Storme

**Affiliations:** Cancer Research Unit, Department of Radiotherapy, Oncology Center, Academic Hospital, Free University Brussels, 1090 Brussels, Belgium

**Keywords:** hypoxia, radiosensitization, *S*-nitroso-*N*-acetylpenicillamine, nitric oxide

## Abstract

The radiosensitizing activity of *S*-nitroso-*N*-acetylpenicillamine (SNAP), a nitric oxide (NO) donor, was assessed in a model of non-metabolic hypoxia achieved in an atmosphere of 95% nitrogen–5% carbon dioxide. A 10 min preincubation of hypoxic EMT-6 cells (10 × 10^6^ ml^−1^) with 0.1 and 1 mM SNAP before radiation resulted in an enhancement ratio of 1.6 and 1.7 respectively. The level of spontaneous NO release, measured by a NO specific microsensor, correlated directly with the concentration of SNAP and was enhanced 50 times in the presence of cells. Dilution of the cell suspension from 10 to 0.1 × 10^6^ ml^−1^ resulted in a 16-fold decline in NO release, but only a twofold decrease in radiosensitization was observed. Preincubation of hypoxic cells with SNAP for 3 min up to 30 min caused an increasing radiosensitizing effect. Extended preincubation of 100 min led to the loss of radiosensitization although the half-life of SNAP is known to be 4–5 h. Taken together, these observations suggest that SNAP generates NO predominantly by a bioreductive mechanism and that its biological half-life is unlikely to exceed 30 min. The lack of correlation between free NO radical and radiosensitizing activity may reflect a role of intracellular NO adducts which could contribute to radiosensitization as well. © 1999 Cancer Research Campaign


*British Journal of Cancer* (1999) **79**(7/8), 1085–1089
© 1999 Cancer Research Campaign
Article no. bjoc.1998.0173

### Radiosensitization of hypoxic tumour cells by *S*-nitroso-*N*-acetylpenicillamine implicates a bioreductive mechanism of nitric oxide generation

**MY Janssens, VN Verovski, DL Van den Berge, C Monsaert and GA Storme**

Cancer Research Unit, Department of Radiotherapy, Oncology Center, Academic Hospital, Free University Brussels, 1090 Brussels, Belgium

**Summary** The radiosensitizing activity of *S*-nitroso-*N*-acetylpenicillamine (SNAP), a nitric oxide (NO) donor, was assessed in a model of non-metabolic hypoxia achieved in an atmosphere of 95% nitrogen–5% carbon dioxide. A 10 min preincubation of hypoxic EMT-6 cells ($10 \times 10^6$ ml$^{-1}$) with 0.1 and 1 mM SNAP before radiation resulted in an enhancement ratio of 1.6 and 1.7 respectively. The level of spontaneous NO release, measured by a NO specific microsensor, correlated directly with the concentration of SNAP and was enhanced 50 times in the presence of cells. Dilution of the cell suspension from 10 to $0.1 \times 10^6$ ml$^{-1}$ resulted in a 16-fold decline in NO release, but only a twofold decrease in radiosensitization was observed. Preincubation of hypoxic cells with SNAP for 3 min up to 30 min caused an increasing radiosensitizing effect. Extended preincubation of 100 min led to the loss of radiosensitization although the half-life of SNAP is known to be 4–5 h. Taken together, these observations suggest that SNAP generates NO predominantly by a bioreductive mechanism and that its biological half-life is unlikely to exceed 30 min. The lack of correlation between free NO radical and radiosensitizing activity may reflect a role of intracellular NO adducts which could contribute to radiosensitization as well.

**Keywords:** hypoxia; radiosensitization; *S*-nitroso-*N*-acetylpenicillamine; nitric oxide

Nitric oxide (NO) donors have recently been evaluated as radiosensitizers of hypoxic cells in a model of metabolism-induced hypoxia at high cell densities. Mitchell et al (1993, 1996) demonstrated that 2-(N,N,-diethylamino)-diazenolate-2-oxide-Na$^+$ (DEA/NO), SNAP and *S*-nitroso-L-glutathione (GSNO) at a concentration of 1 mM, radiosensitized Chinese hamster V79 lung fibroblasts to a similar extent as oxygen. Griffin et al (1996) reported comparable activity for DEA/NO and (Z)-1-{N-[3-aminopropyl]-N-[4-(3-aminopropylammonio)butyl-amino}-diazen-1-ium-1,2-diolate (SPER/NO) with enhancement ratios of 2.8–3.0 in SCK mammary carcinoma cells exposed to 1–2 mM radiosensitizer. Our laboratory investigated the radiosensitizing activity of SNP in a panel of eight human pancreatic tumour cell lines and found an overall enhancement ratio of 1.9 at 0.1 mM (Verovski et al, 1996). At 0.3–1 mM, SNP caused almost complete radiosensitization in hypoxic PSN1/ADR cells that was accompanied by restoration of radiation-induced DNA breakage up to the level in aerated cells.

These data collectively confirm the high radiosensitizing efficiency of NO donors at 1–2 mM and suggest a role of NO in the fixation of DNA damage caused by radiation. Whether DNA is the main target for NO, as was postulated by Howard-Flanders (1957), or whether other mechanisms contribute to the enhanced DNA damage remains unclear. As NO can interact with iron–sulphur containing enzymes, this may result in the inhibition of cellular respiration and sparing of the natural sensitizer oxygen (Mitchell et al, 1996). This sensitizing effect mediated by residual oxygen can be minimized under hypoxic conditions induced by nitrogen gassing, a general approach to study radiosensitizers. Moreover, the latter model of hypoxia allows the use of a broad range of cell densities, which is a necessary step to establish whether the rate of NO release is enhanced by the presence of cells. The NO donors DEA/NO and SPER/NO are known to liberate NO by a purely spontaneous mechanism (Maragos et al, 1991). Some NO donors, such as SNAP and GSNO, have generally been assumed to release NO in a spontaneous manner although the slow decomposition rate of *S*-nitrosothiols appeared to underestimate their biological effects (Kowaluk and Fung, 1990). In fact, the chemistry of *S*-nitrosothiols containing NO as a nitrosonium cation (Stamler et al, 1992), supports the idea of reductive catalysis of NO release, which may occur on the cellular membrane (Bates et al, 1991; Kowaluk et al, 1990; Rochelle et al, 1994). In the report of Mitchell et al (1996), the possibility of bioreductive generation of NO from SNAP has not been explored, but NO output in a cell-free system was apparently too low to account for radiosensitization. The same conclusion was drawn for another nitrosonium-like NO donor, SNP, whose bioreductive activation was found to be responsible for radiosensitization (Verovski et al, 1996).

The objective of the present study was to examine whether bioreductive release of NO from SNAP is implicated in hypoxic cell radiosensitization. To verify this hypothesis we compared the rate of NO release in the absence or presence of cells and related NO measurements to radiosensitizing concentrations of SNAP in hypoxia. Nitrogen gassing was chosen as a model of hypoxia because it allows radiosensitizing experiments to be performed at low cell densities and its use excludes possible sensitizing effects due to oxygen sparing.

*Received 2 December 1997*
*Revised 10 April 1998*
*Accepted 30 June 1998*

*Correspondence to:* MY Janssens, Department of Radiotherapy, Oncology Center, A.Z.-V.U.B., Laarbeeklaan 101, 1090 Brussels, Belgium

### MATERIALS AND METHODS

### Chemicals

SNAP, (2)-1-[N-(3-ammoniopropyl)-N-(n-propyl)amino)diazen-1-ium-1,2-diolat (PAPA/NO) and 2-(4-carboxyphenyl)-4,4,5,5-tetramethylimidazoline-1-oxyl-3-oxide (carboxy-PTIO) were purchased from Alexis Corporation (Laufelfingen, Switzerland). Other chemicals were obtained from Sigma Chemical Co. (St. Louis, MO, USA). The stocks of SNAP and PAPA/NO were prepared in dimethylsulphoxide (DMSO) and 0.01 M sodium hydroxide respectively.

### Cell culture

Murine mammary adenocarcinoma EMT-6 cells were cultured in RPMI-1640 medium (Gibco, Paisley, UK) supplemented with 10% bovine calf serum (HyClone Laboratories Logan, UT, USA) at 37°C in 5% carbon dioxide/95% air.

### Radiation

Cultures grown to early confluence were trypsinized, and the cells were washed by centrifugation in medium and counted. All steps in sample preparation and processing were performed at 0°C unless otherwise stated. Hypoxia in a cell suspension was achieved by repeated vacuum evacuation/injection (60/30 s) of 5% carbon dioxide/95% nitrogen for 30 min. Hypoxic cells ($0.1–10 \times 10^6$ ml$^{-1}$ in 150 µl) placed in glass tubes were preincubated for 10 min at 37°C with or without radiosensitizer before radiation. This preparatory procedure did not significantly alter pH, which ranged from 7.2 to 7.4 depending on cell density. In a separate set of experiments, the preincubation time varied between 3 and 100 min. Metabolic hypoxia in cell pellets was achieved as described previously (Verovski et al, 1996). Briefly, SNAP was added at appropriate concentrations to cells ($0.5 \times 10^6$ in 100 µl of medium) kept in conical plastic tubes on ice. Pellets of approximately 0.3–0.4 mm in height were produced by centrifugation at 300 ***g*** for 5 min. Metabolic oxygen depletion was induced prior to radiation by a 10-min incubation at 37°C. Cells in both suspensions and pellets were irradiated at 37°C at a dose rate of 2 Gy min$^{-1}$ using an 8-MV photon beam from a linear accelerator and immediately cooled. The survival of control and irradiated cells was measured by an 8-day colony formation assay as described previously (Verovski et al, 1996). To analyse cellular radiosensitivity, radiation survival curves were produced after linear–quadratic fitting of the dose–survival data. The radiation doses involved were 2, 4, 6 and 8 Gy for oxic cells and 4, 8, 12 and 16 Gy for hypoxic cells. The enhancement ratios for oxygen and SNAP were calculated at the level of 0.1 survival fraction by dividing the radiation dose of hypoxic cells by the radiation dose of oxic cells (or hypoxic cells plus radiosensitizer).

### Amperometric measurement of nitric oxide

All measurements were conducted in open vials, at 37°C and with periodic gentle stirring. Spontaneous NO release from 0.1–10 mM SNAP was estimated in the absence of cells. The NO signal was allowed to stabilize for 5 min, after which SNAP was added to the final concentration. Starting from 6 min, the NO signal was registered every minute by an Iso-NOP200 microsensor connected to an ISO-NO Mark II meter (both from World Precision Instruments, Hertfordshire, UK). The amperometric measurements were expressed in nA. To assess bioreductive generation of NO, SNAP at 0.1 mM was incubated in medium containing EMT-6 cells ($0.1–10 \times 10^6$ ml$^{-1}$), and NO measurements were performed as described above. When used, the NO-specific scavenger carboxy-PTIO was injected prior to SNAP to produce a final concentration of 0.3 mM.

### Statistics

All assays were repeated at least three times. Data are expressed as means (symbols) with corresponding standard deviations (bars).

### RESULTS

### Amperometric measurement of NO generated from SNAP

The NO release profiles from SNAP in the absence or presence of EMT-6 cells are summarized in Figure 1. In a cell-free system (Figure 1A), the NO output decreased 16- and 160-fold with the dilution of SNAP from 10 to 1 and 0.1 mM respectively. At 0.1 mM SNAP, addition of EMT-6 cells progressively activated the NO release up to 50 times at $10 \times 10^6$ cells ml$^{-1}$ (Figure 1B). To ascertain the specificity of the NO sensor under the conditions used, the NO scavenger carboxy-PTIO was added to the cells ($10 \times 10^6$ ml$^{-1}$) before injection of SNAP. This led to a complete loss of the NO signal.

### Radiosensitizing properties of SNAP

The radiosensitizing activity of SNAP was assessed at 0.1 mM, a concentration that allows us to focus on bioreductive NO generation because of the low background of spontaneous NO release. The radiation survival curves of EMT-6 cells in normoxia, and hypoxia with or without radiosensitizer are shown in Figure 2. The enhancement ratios for SNAP at cell densities of 0.1, 1 and $10 \times 10^6$ ml$^{-1}$ were 1.3, 1.4 and 1.6 respectively, which did not reflect the considerable changes in NO profiles demonstrated in Figure 1. The lack of correlation between the NO signal and radiosensitization at different cell densities was not due to the variations in hypoxia, which was forced by nitrogen gassing. The oxygen enhancement ratios were identical (2.8–2.9) and the radiosensitivity of hypoxic cells was similar for all three cell densities. The aerobic survival curves did not indicate any shift in radiosensitivity thus confirming the absence of metabolic oxygen depletion. Only above $10 \times 10^6$ ml$^{-1}$ did aerated cells reveal significant radioprotection due to metabolic oxygen depletion (data not shown), and therefore these conditions were excluded from analysis of both radiosensitivity and NO release. We tested the effect of carboxy-PTIO, an NO scavenger, on the radiosensitizing activity of SNAP at a cell density of $10 \times 10^6$ ml$^{-1}$. Half-reversal of radiosensitization was observed although complete scavenging of the extracellular NO was confirmed by the NO microsensor (see Figure 1B). Using the same cell density, we further investigated the radiosensitizing activity of SNAP in a range of concentrations between 0.01 and 1 mM. Figure 3 summarizes the enhancement ratios of different concentrations of SNAP calculated from survival curves (not shown). We observed an increase of the enhancement ratios with rising concentrations of SNAP, levelling out at 1.6–1.7.

 © Cancer Research Campaign 1999

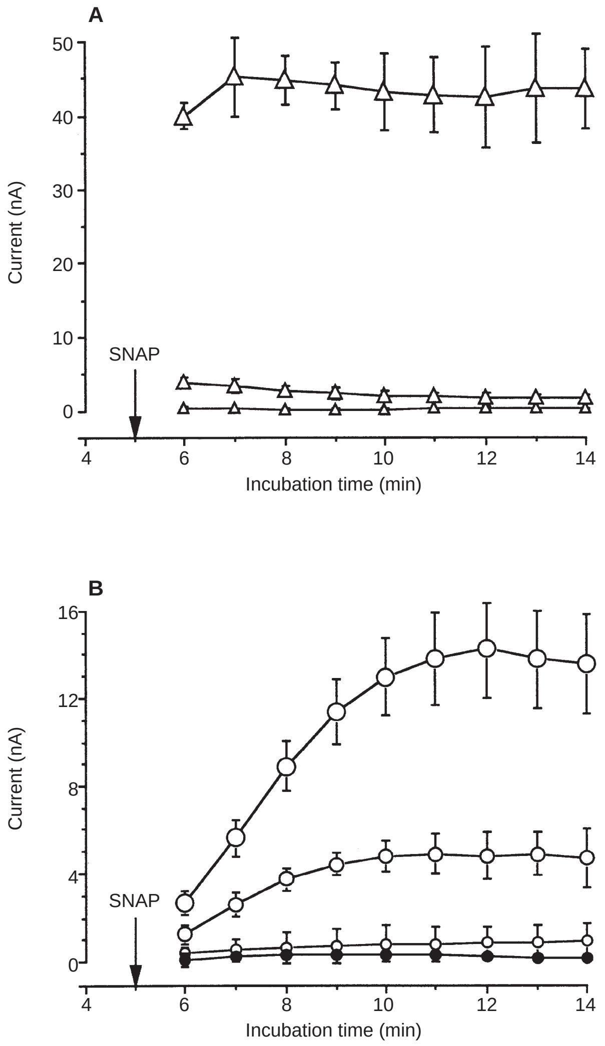

**Figure 1** Amperometric measurement of NO released from SNAP at 37°C in the absence (**A**) or in the presence (**B**) of EMT-6 cells. The NO signal was allowed to stabilize during 5 min and afterwards SNAP was added as indicated by the arrows. (**A**) SNAP was incubated at concentrations of 0.1 (△), 1 (△) and 10 mM (△). (**B**) SNAP (0.1 mM) was incubated in a cell suspension containing $0.1 \times 10^6$ (○), $1 \times 10^6$ (○) and $10 \times 10^6$ (○) cells $ml^{-1}$. The NO scavenger carboxy-PTIO was added to the cells ($10 \times 10^6$ $ml^{-1}$) immediately before SNAP (●)

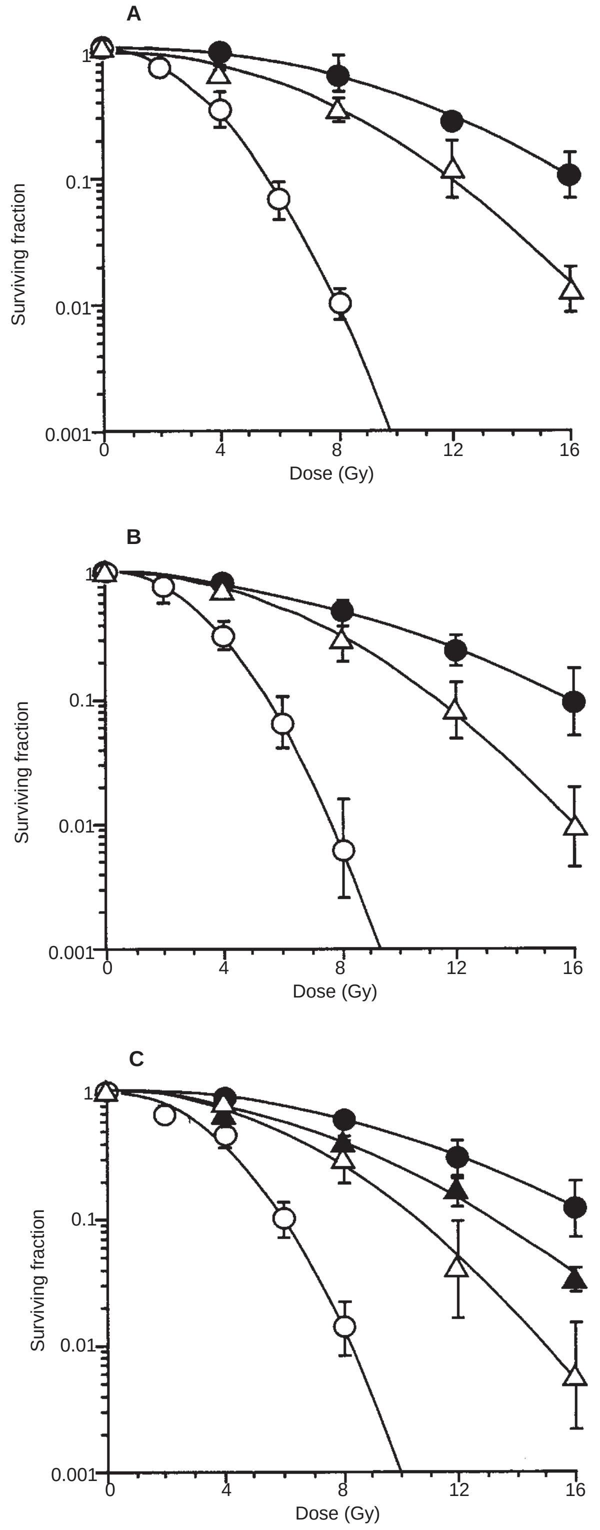

**Figure 2** Radiosensitizing activity of SNAP (0.1 mM) in EMT-6 cells at cell densities of 0.1 (**A**), 1 (**B**) and 10 (**C**) $\times 10^6$ $ml^{-1}$ in a model of nitrogen-induced hypoxia. Cell survival in normoxia (○), hypoxia (△), hypoxia + SNAP (●) or hypoxia + SNAP + carboxy-PTIO (▲) was estimated by a colony formation assay

It is worthy of note that the maximal radiosensitizing effect of SNAP described here is significantly less than the data of Mitchell et al (1996), who found an enhancement ratio of 2.6 for 1 mM SNAP in V79 lung fibroblasts metabolically depleted of oxygen. Therefore, the activity of SNAP was reevaluated in cell pellets, a model of metabolic hypoxia previously applied to SNP (Verovski et al, 1996). When compared with nitrogen-gassed cell suspensions, freely aerated pellets provided a decreased level of hypoxia with an oxygen enhancement ratio of 2.5 (Figure 4). No significant change in the enhancement ratios (1.5–1.9) for 0.1–1 mM SNAP was observed.

### Bioactivation rate of SNAP in hypoxia

As NO measurements had indicated fast decomposition of SNAP in the presence of cells (Figure 1), we decided to investigate the

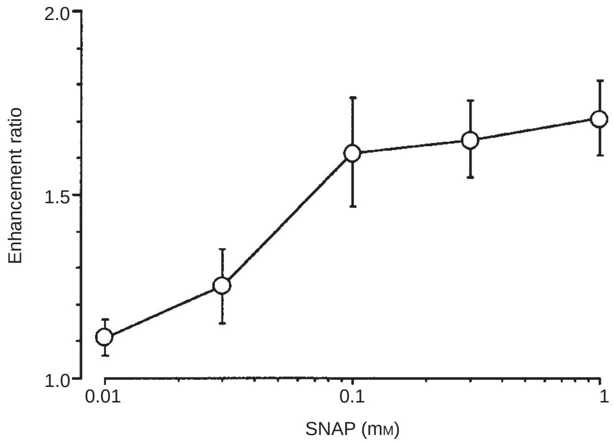

**Figure 3** Concentration dependency of the radiosensitizing activity of SNAP. Hypoxic EMT-6 cells (10 × $10^6$ $ml^{-1}$) were exposed to 0.01–1 mM SNAP for 10 min prior to radiation and the enhancement ratio was calculated from the surviving curves

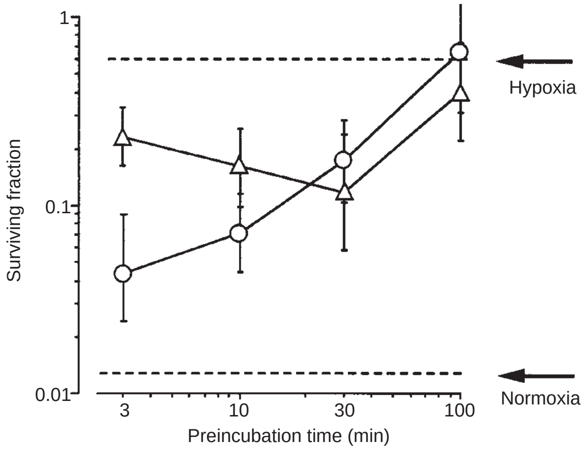

**Figure 5** Radiosensitizing activity of SNAP (△) and PAPA/NO (○) in hypoxic EMT-6 cells as a function of preincubation time. Cells (10 × $10^6$ $ml^{-1}$) were preincubated during 3–100 min with 0.3 mM radiosensitizer, irradiated at 8 Gy and analysed for survival. The cell survival without radiosensitizer in normoxia and hypoxia is indicated by arrows

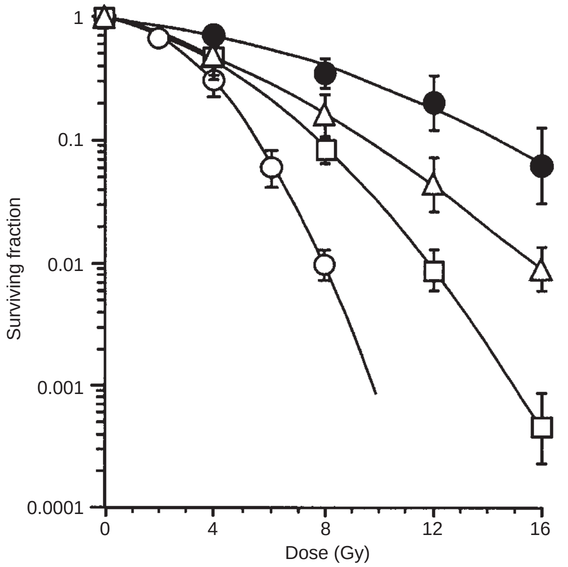

**Figure 4** Radiosensitizing activity of SNAP in EMT-6 cells in a model of metabolism-induced hypoxia in pellets. Cells were irradiated in absence (●) or presence of SNAP at 0.1 mM (△) or 1 mM (□) and cell survival was estimated by a colony formation assay. The cell survival curve in normoxia (○) in cell suspensions from Figure 2A was also plotted for reference bioactivation rate of SNAP in hypoxia with regard to radiosensitizing activity. EMT-6 cells were preincubated with the radiosensitizer for different time periods up to 100 min and the cell survival at 8 Gy was estimated (Figure 5). Between 3 and 30 min, an increase in radiosensitization was observed followed by a significant decline at 100 min. A similar decline in radiosensitization was found for the NO donor PAPA/NO, which is known to undergo decomposition in a purely spontaneous way with a half-life of 15 min (Maragos et al, 1991). Therefore the biological half-life of SNAP is unlikely to exceed 30 min.

### DISCUSSION

In the present study, the role of bioreductive NO release from SNAP in hypoxic cell radiosensitization was evaluated. Hypoxia was induced by nitrogen gassing because radiosensitization was to be performed at low cell densities. Hypoxic EMT-6 cells treated with SNAP in a broad range of cell (0.1–10 × $10^6$ $ml^{-1}$) and radiosensitizer (0.01–1 mM) concentrations clearly showed increased response to radiation with a maximal enhancement ratio of 1.7. This activity is far less than the observation of Mitchell et al (1996), who used V79 lung fibroblasts in a model of metabolic hypoxia and reported enhancement ratios of 2.5–2.6 for 0.1–1 mM SNAP. With the same approach, many other NO donors (DEA/NO, SPER/NO, GSNO, SNP) were investigated, and in some but not all cell lines enhancement ratios close to that of oxygen (2.6–3.0) were observed (Mitchell et al, 1993, 1996; Griffin et al, 1996; Verovski et al, 1996).

The reduced activity of SNAP in EMT-6 cells may reflect a diminished sensitivity of these cells to NO or, alternatively, could be associated with the use of nitrogen gassing to induce hypoxia. To check out the latter possibility, EMT-6 cells were metabolically depleted of oxygen in cell pellets, a method previously applied to sodium nitroprusside (SNP) (Verovski et al, 1996). In these conditions, SNAP demonstrated a maximal enhancement ratio of 1.9, which is still less than that (2.5–2.6) found in V79 lung fibroblasts (Mitchell et al, 1996). Therefore, we believe that the most likely explanation for this discrepancy is a cell-dependent variation in the activity of SNAP. Significant variability in the radiosensitizing effect of the bioreductive NO donor SNP was described earlier in human pancreatic cell lines (Verovski et al, 1996). Such a phenomenon might be especially prominent for bioreductive agents whose catalytic transformations are expected to be cell specific. We speculated that the release of NO from SNAP proceeds in a bioreductive way that is analogous to SNP, and hence SNAP may also reveal a variable radiosensitizing potential. We focus further analysis on the hypothesis of a bioreductive mechanism of NO generation from SNAP; while the evaluation of its radiosensitizing potency with regard to different cell lines and hypoxia models is a matter for future experiments.

Using a NO specific microsensor, we measured the release of NO from SNAP in the absence or presence of EMT-6 cells, and the corresponding mechanisms were referred to as spontaneous and bioreductive. This interpretation does not imply intracellular

 © Cancer Research Campaign 1999

transformations through reductases known to activate classical bioreductive cytotoxines (Adams, 1992), because highly polar S-nitrosothiols are unlikely to penetrate biological membranes easily (Kowaluk et al, 1990). Instead, activation at the cellular membrane resulting in reductive release of NO or its direct transfer towards thiols and other nucleophilic targets is being considered (Bates et al, 1991; Kowaluk et al, 1992; Rochelle et al, 1994). We found that the generation of NO from SNAP was strongly dependent on cell density, indicating bioreductive catalysis to be a predominant mechanism of NO release. Actually, spontaneous liberation of NO was only significant at a concentration of 1.0 mM SNAP or more, in line with the data of Mitchell et al (1996). The addition of cells up to $10 \times 10^6$ ml$^{-1}$ resulted in a 50-fold increase in the NO signal, which became apparent at 0.1 mM SNAP, a concentration found to cause radiosensitization. The level of NO release, however, did not correspond to the radiosensitizing activity, as also observed by Mitchell et al (1996). A 16-fold decline in the NO signal was observed after dilution of the cell suspension to $0.1 \times 10^6$ ml$^{-1}$, whereas radiosensitization decreased only twofold. In addition, only half-reversal in SNAP-induced radiosensitization was obtained in the presence of the NO scavenger carboxy-PTIO, though the NO signal disappeared completely. Conceivably, the radiosensitization observed despite a very low NO signal may be attributed to the intracellular NO pool, which cannot be detected by the NO sensor nor scavenged by carboxy-PTIO. This agent is believed not to cross the cell membrane and would therefore not be capable of neutralizing the intracellular NO, which exists primarily in the form of *S*-nitrosothiols. In particular, intracellular glutathione is thought to be an important intermediary of the bioactive NO pool (Ignarro et al, 1981; Clancy et al, 1994), consistent with the radiosensitizing properties of GSNO (Mitchell et al, 1996; Verovski et al, 1996).

We also investigated the impact of timing on radiosensitization because NO measurements has shown accelerated decomposition of SNAP in the presence of cells. The experimental protocols to preincubate hypoxic cells with NO donors vary in different reports, and a time period between 2 to 10 half-lives was arbitrarily taken, aiming at maximization of the NO level (Mitchell et al, 1993, 1996; Griffin et al, 1996; Verovski et al, 1996). This approach is difficult to apply to SNAP, a chemical with a half-life of more than 4 h (Ignarro et al, 1981). A loss of radiosensitizing activity between 30 and 100 min preincubation time was found in a manner close to the radiosensitizing profile of PAPA/NO, a NO donor with a half-life of 15 min. Therefore, the biological half-life of SNAP is unlikely to exceed 30 min at a cell density of $10 \times 10^6$ ml$^{-1}$. An accelerated decomposition of *S*-nitrosothiols under physiological conditions has already been suggested in the literature to explain the discrepancy between their chemical and biological properties (Kowaluk et al, 1992). Conceivably, not only the presence of cells but also hypoxic conditions may influence the stability of SNAP. The latter possibility is supported by the data of Ioannidis et al (1996), who found a faster decomposition and higher cytotoxicity of SNAP in hypoxia compared with aerobic conditions.

In conclusion, the negligible rate of SNAP decomposition in a cell-free system argues against the role of spontaneous NO liberation in hypoxic cell radiosensitization. On the contrary, bio-reductive NO generation from SNAP at radiobiologically active concentrations is an order of magnitude higher and is therefore more likely to sustain radiosensitization.

### ACKNOWLEDGEMENTS

This research was funded by grants nos 3.0036.94, G.0055.96 and G.0195.98 from the Fonds voor Wetenschappelijk Onderzoek-Vlaanderen, Sportvereniging tegen Kanker and Vlaamse Liga tegen Kanker.

### ABBREVIATIONS

carboxy-PTIO, 2-(4-carboxyphenyl)-4,4,5,5-tetramethylimidazoline-1-oxyl-3-oxide; DEA/NO, 2-(N,N-diethylamino)-diazenolate-2-oxide-$Na^+$; GSNO, S-nitroso-L-glutathione; NO, nitric oxide; PAPA/NO, (2)-1-[N-(3-ammoniopropyl)-N-(n-propyl)amino)diazen-1-ium-1,2-diolate]; SNAP, S-nitroso-N-acetylpenicillamine; SNP, sodium nitroprusside; SPER/NO, (Z)-1-{N-[3-aminopropyl]-N-[4-(3-aminopropylammonio)butyl-amino}-diazen-1-ium-1,2-diolate

### REFERENCES

Adams GE (1992) Redox, radiation, and reductive bioactivation. *Radiat Res* **132**: 129–139

Bates JN, Baker MT, Guerra R and Harrison DG (1991) Nitric oxide generation from nitroprusside by vascular tissue. Evidence that reduction of the nitroprusside anion and cyanide loss are required. *Biochem Pharmacol* **42**: S157–S165

Clancy RM, Levartovsky D, Leszczynska-Piziak J, Yegudin J and Abramson SB (1994) Nitric oxide reacts with intracellular glutathione and activates the hexose monophosphate shunt in human neutrophils: evidence for S-nitrosoglutathione as a bioactive intermediary. *Proc Natl Acad Sci USA* **91**: 3680–3684

Griffin RJ, Makepeace CM, Hur WJ and Song CW (1996) Radiosensitization of hypoxic tumor cells in vitro by nitric oxide. *Int J Rad Onc Biol Phys* **36**: 377–383

Howard-Flanders P (1957) Effect of nitric oxide on the radiosensitivity of bacteria. *Nature* **180**: 1191–1192

Ignarro LJ, Lippton H, Edwards JC, Baricos WH, Hyman AL, Kadowitz PJ and Gruetter CA (1981) Mechanism of vascular smooth muscle relaxation by organic nitrates, nitrites, nitroprusside and nitric oxide: evidence for the involvement of S-nitrosothiols as active intermediates. *J Pharm Exp Ther* **218**: 739–749

Ioannidis I, Batz M, Paul T, Korth H, Sustmann R and Groot H (1996) Enhanced release of nitric oxide causes increased cytotoxicity of S-nitroso-n-acetyl-DL-penicillamine and sodium nitroprusside under hypoxic conditions. *Biochem J* **318**: 789–795

Kowaluk EA and Fung HL (1990) Spontaneous liberation of nitric oxide cannot account for in vitro vascular relaxation by S-nitrosothiols. *J Pharm Exp Ther* **255**: 1256–1264

Maragos CM, Morley D, Wink D, Dunams TM, Saavedra JE, Hoffman A, Bove AA, Isaac L, Hrabie JA and Keefer LK (1991) Complexes of NO with nucleophiles as agents for the controlled biological release of nitric oxide. Vasorelaxant effects. *J Med Chem* **34**: 3242–3247

Mitchell JB, Wink DA, DeGraff W, Gamson J, Keefer LK and Krishna MC (1993) Hypoxic mammalian cell radiosensitization by nitric oxide. *Cancer Res* **53**: 5845–5848

Mitchell JB, Cook JA, Krishna MC, DeGraff W, Gamson J, Fisher J, Christodoulou D and Wink DA (1996) Radiation sensitisation by nitric oxide releasing agents. *Br J Cancer* **74**: suppl. XXVII, S181–S184

Rochelle LG, Kruszyna H, Kruszyna R, Barchowsky A, Wilcox DE and Smith RP (1994) Bioactivation of nitroprusside by porcine endothelial cells. *Toxicol Appl Pharmacol* **128**: 123–128

Stamler JS, Singel DJ and Loscalzo J (1992) Biochemistry of nitric oxide and its redox-activated forms. *Science* **258**: 1898–1902

Verovski VN, Van den Berge DL, Soete GA, Bols BL and Storme GA (1996) Intrinsic radiosensitivity of human pancreatic tumour cells and the radiosensitising potency of the nitric oxide donor sodium nitroprusside. *Br J Cancer* **74**: 1734–1742

© Cancer Research Campaign 1999
